# Draft Genome Sequence of *Ralstonia* sp. Strain GA3-3, Isolated from Australian Suburban Soil

**DOI:** 10.1128/genomeA.00414-13

**Published:** 2013-07-05

**Authors:** Stephen L. Pearce, Hafizah Pushiri, John G. Oakeshott, Robyn J. Russell, Gunjan Pandey

**Affiliations:** CSIRO Ecosystem Sciences, Acton, Australian Capital Territory, Australiaa; Research School of Chemistry, Australian National University, Australian Capital Territory, Australiab; Faculty of Environmental Studies, Universiti Putra Malaysia, Serdang, Malaysiac

## Abstract

*Ralstonia* sp. strain GA3-3 is a hexachlorocyclohexane (HCH)-degrading bacterial strain isolated from suburban soil in Canberra, Australia. The genome of strain GA3-3 was sequenced to investigate its ability to degrade α-HCH. Here, we report the annotated genome sequence of this strain.

## GENOME ANNOUNCEMENT

Several bacterial strains were isolated during a hexachlorocyclohexane (HCH) degradation study in Australia. Two strains, including *Ralstonia* sp. strain GA3-3, were chosen to be sequenced based on their HCH degradation phenotype. GA3-3 was isolated from suburban soil with no known history of HCH exposure in Canberra, Australia, after three cycles of enrichment with 50 ppm α-HCH as the sole carbon source. Further study of GA3-3 revealed that it was able to degrade α-HCH, albeit incompletely and very slowly compared to other known HCH degraders (1).

The genomic DNA of GA3-3 was prepared using the Qiagen Genomic-tip 20/G kit for bacteria, according to the manufacturer’s instructions. Fragments of 500-bp length were then sequenced using Illumina HiSeq 2000 technology at the John Curtin School of Medical Research, Australian National University. The Ray assembler was used to assemble 8,533,778 100-bp paired-end reads using a *k*-mer length of 63 (2). This assembly generated 62 contigs of >500 bp in length, with an N_50_ value of 193,594 bp. The total size of the assembly was 6.7 Mb, with a G+C content of 66.7%. The paired-end information was able to combine 26 of the contigs into 11 scaffolds, making a total of 47 scaffolds or contigs for the assembly.

Annotation of the genome using the NCBI Prokaryotic Genomes Automatic Annotation Pipeline (PGAAP) predicted 6,174 protein-coding sequences in GA3-3, with 1,439 (23.4%) annotated only as hypothetical proteins. The assembly was also predicted to contain 55 tRNA and 14 rRNA sequences. A BLAST search of the GA3-3 16S rRNA sequence against the NCBI database revealed 99.7% (1,403-nucleotide alignment) identity between GA3-3 and other *Ralstonia* spp.; however, further investigation is needed to determine the true identity of GA3-3.

All previously studied HCH-degrading bacteria share the same pathway for HCH degradation, which requires the *linA-linF* genes (3–6). A BLAST search was performed on the genome of GA3-3 using the protein sequences encoded by the *linA-linF* genes (accession no. YP_003545302, BAI96793, YP_003544005, YP_003547114, YP_003547119, and BAI98845, for *linA*, *linB*, *linC*, *linD*, *linE*, and *linF*, respectively) of *Sphingobium japonicum* UT26S from the NCBI database. However, no *linA-linF* genes or their homologues were detected in GA3-3 (1). Another α-HCH degradation study that was performed using GA3-3 at this stage revealed no degradation activity (S. Pearce, unpublished data). The absence of these genes, coupled with the knowledge that the *lin* genes are normally associated with the transposable insertion element IS*6100* (5, 7, 8), may explain the loss of activity since the first degradation experiment.

### Nucleotide sequence accession numbers.

This whole-genome shotgun project has been deposited at DDBJ/EMBL/GenBank under the accession no. AQPZ00000000. The version described in this paper is version AQPZ01000000.

## References

[1] Mohd Pushiri NH 2012 M Biotech (Research) thesis Australian National University, Canberra, Australia

[2] Boisvert S Laviolette F Corbeil J 2010 Ray: simultaneous assembly of reads from a mix of high-throughput sequencing technologies. J. Comput. Biol. 17:1519–1533 20958248 10.1089/cmb.2009.0238 PMC3119603

[3] Lal R Pandey G Sharma P Kumari K Malhotra S Pandey R Raina V Kohler HP Holliger C Jackson C Oakeshott JG 2010 Biochemistry of microbial degradation of hexachlorocyclohexane and prospects for bioremediation. Microbiol. Mol. Biol. Rev. 74:58–80 20197499 10.1128/MMBR.00029-09 PMC2832351

[4] Singh AK Chaudhary P Macwan AS Diwedi UN Kumar A 2007 Selective loss of *lin* genes from hexachlorocyclohexane-degrading *Pseudomonas aeruginosa* ITRC-5 under different growth conditions. Appl. Microbiol. Biotechnol. 76:895–901 17602219 10.1007/s00253-007-1056-z

[5] Dogra C Raina V Pal R Suar M Lal S Gartemann KH Holliger C van der Meer JR Lal R 2004 Organization of *lin* genes and IS*6100* among different strains of hexachlorocyclohexane-degrading *Sphingomonas paucimobilis*: evidence for horizontal gene transfer. J. Bacteriol. 186:2225–2235 15060023 10.1128/JB.186.8.2225-2235.2004 PMC412113

[6] Kumari R Subudhi S Suar M Dhingra G Raina V Dogra C Lal S van der Meer JR Holliger C Lal R 2002 Cloning and characterization of *lin* genes responsible for the degradation of hexachlorocyclohexane isomers by *Sphingomonas paucimobilis* strain B90. Appl. Environ. Microbiol. 68:6021–6028 12450824 10.1128/AEM.68.12.6021-6028.2002 PMC134425

[7] Böltner D Moreno-Morillas S Ramos JL 2005 16S rDNA phylogeny and distribution of *lin* genes in novel hexachlorocyclohexane-degrading *Sphingomonas* strains. Environ. Microbiol. 7:1329–1338 16104856 10.1111/j.1462-5822.2005.00820.x

[8] Lal R Dogra C Malhotra S Sharma P Pal R 2006 Diversity, distribution and divergence of *lin* genes in hexachlorocyclohexane-degrading sphingomonads. Trends Biotechnol. 24:121–130 16473421 10.1016/j.tibtech.2006.01.005

